# Patients’ self-reported receipt of brief smoking cessation interventions based on a decision support tool embedded in the healthcare information system of a large general hospital in China

**DOI:** 10.18332/tid/112567

**Published:** 2019-10-18

**Authors:** Shuilian Chu, Lirong Liang, Hang Jing, Di Zhang, Zhaohui Tong

**Affiliations:** 1 Department of Clinical Epidemiology & Tobacco Dependence Treatment Research, Beijing Chaoyang Hospital, Capital Medical University, Beijing, China; 2 Beijing Institute of Respiratory Medicine, Beijing, China; 3 Department of Respiratory and Critical Care Medicine, Beijing Chaoyang Hospital, Capital Medical University, Beijing, China

**Keywords:** smoking cessation, China, brief intervention, clinician, healthcare information system

## Abstract

**INTRODUCTION:**

Healthcare information systems (HIS) are used to aid healthcare providers delivering brief smoking cessation interventions. However, evidence regarding the effectiveness of intervention models in developing countries remains limited. A smoking cessation intervention model based on a decision support tool embedded in HIS (an ‘e-information model’, including Ask, Advise, Assess, Inform, Refer and Print components) was applied in a large urban general hospital in Beijing, China. The current study was a preliminary evaluation of the implementation and effectiveness of this model.

**METHODS:**

We conducted a retrospective investigation in the outpatient department of the hospital in the period June–July 2017. Using a paper questionnaire, patients’ self-reported receipt of the e-information model in the past 2 months and their plans to quit within 1 month were collected. Multivariate logistic regression analysis was used to examine the association between receiving the e-information model and patients’ plans to quit.

**RESULTS:**

Among 656 currently smoking patients, the proportion of patients receiving the Ask, Advise, Assess, Refer and Print components were 73.2%, 65.4%, 49.8%, 16.0% and 10.4%, respectively. The results revealed a dose-response relationship between the number of components received and the proportion of patients planning to quit (p-trend=0.006). The likelihood of patients planning to quit within 1 month was highest among those receiving all five components (OR=2.79, 95% CI: 1.31–5.94). Moreover, a simplified model composed of two or three components also revealed a potential effect on increasing the proportion of patients planning to quit.

**CONCLUSIONS:**

The e-information model was applied effectively in the study hospital and appeared to encourage patients to plan to quit smoking. This model could be generalized to other hospitals in China and other developing countries. However, many components of this model were less utilized, and comprehensive measures will be required to improve its application in the future.

## INTRODUCTION

Smoking is the leading preventable cause of disability and death in the world. In China, the world’s largest tobacco consumer^1^, more than 1 million people die each year from smoking-related diseases^2^. Smoking cessation is the most effective measure for preventing smoking-related diseases^3^. However, the quit rate among Chinese smokers remains low (2010: 12.8%; 2015: 14.4%)^1,4^, partially explaining why smoking prevalence in China has not declined significantly in recent years (2010: overall 28.1%, men 52.9% and women 2.4% ; 2015: overall 27.7%, men 52 .1% and women 2.7%)^1,4^. Previous evidence indicates that providing cessation assistance to smokers can effectively increase the quit rate^5^. However, the proportion of Chinese smokers receiving cessation assistance is currently very low (only half of Chinese smokers had received quit advice from physicians, and no more than 3% of Chinese smokers had used cessation medications)^4^. In view of this, strengthening smoking cessation services had therefore been highlighted in the outline of the ‘Healthy China 2030’ plan in order to achieve the goal of reducing the smoking rate in Chinese to 20% by 2030^6^.

Healthcare providers (HPs) play an important role in smoking cessation services. Most smokers visit a clinician annually, providing a unique opportunity to identify smokers and provide cessation interventions in the clinic^5^. Given that intensive smoking cessation interventions are time-consuming, brief smoking cessation interventions are more suitable for provision by clinicians, including the 5 As (Ask, Advise, Assess, Assist, Arrange)^5^, 3 As (Ask, Advise, Assist)^7^, ABC (Ask, Brief advice, Cessation support)^8^, and AAR (Ask, Advise, Refer)^9^ models. However, a previous study revealed that most HPs delivered Ask, Advise and Assess interventions frequently, while far fewer HPs provided Assist and Arrange interventions, due to limited time, lack of skills or organizational support^10^. To overcome these difficulties, many researchers have attempted to use healthcare information systems (HIS) to aid HPs in providing cessation interventions, such as electronic health record (EHR), clinical decision support system (CDSS), and electronic prescribing system (EPS)^11^. It has been reported that smoking cessation interventions based on HIS can effectively support HPs to document patients’ smoking status and refer smoking patients to smoking cessation services^11^. Although this kind of intervention model has been applied widely in many developed countries, the evidence regarding the effectiveness of such intervention models in developing countries, particularly China, remains limited. In 2009, a brief smoking cessation intervention model called the e-information model (including Ask, Advise, Assess, Inform, Refer and Print components) based on a decision support tool embedded in the HIS was developed and applied in a large urban general hospital in Beijing, China. This initiative represents the first attempt to apply a brief smoking cessation intervention based on HIS changes in a developing country. This hospital was chosen to pilot the project due to its outstanding clinical and research capability in smoking cessation services as a result of being the first hospital to set up a smoking cessation clinic in China in 1996 and providing a free national smoking cessation hotline in mainland China in 2004^12^. The purpose of the current study was to conduct a preliminary evaluation of the implementation and effectiveness of the e-information model, to identify limitations or obstacles to optimizing the model, and to then generalize it to other general hospitals in China and as well as those in other developing countries in the future.

## METHODS

### Study design

This was a retrospective investigation conducted in the outpatient department of a large urban general hospital in Beijing, China, in the period June–July 2017. In China, outpatient departments are an important part of hospitals and are composed of many specialist clinics according to the diseases treated. We chose three clinics in the outpatient departments of the hospital, including Respiratory, Cardiology and Neurology clinics, with a high volume of patients, and in which most patients were current smokers suffering from smoking-related diseases such as chronic obstructive pulmonary disease, cardiocerebral vascular diseases and stroke. These patients are the target population of the e-information model. Patients’ self-reported responses regarding whether they received the components of the e-information model in the past 2 months and whether they planned to quit smoking within 1 month were collected via a paper questionnaire. The study protocol was approved by the Ethics Committee of the hospital. All participants provided informed consent for their voluntary participation.

### Participants

The investigation was conducted Monday to Friday, 8 am to 5 pm, between June and July 2017. Three trained staff recruited the eligible patients and conducted the questionnaire in the waiting areas of the respiratory, cardiology and neurology clinics before patients visited the clinicians. We selected participants who were older than 18 years of age, had visited any of the three clinics in the past 2 months, had smoked 100 or more cigarettes in their lives, and currently smoked every day. Patients who reported non-daily smoking or who were unwilling to provide individual demographic information were excluded.

**Figure 1:**
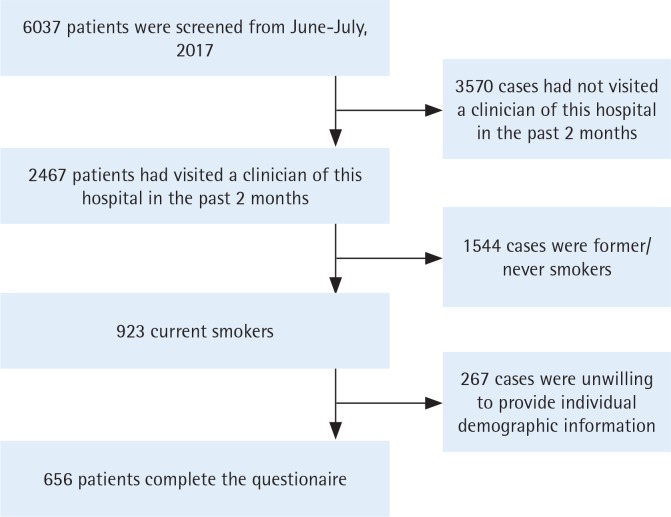
Flow chart of screening and enrollment of eligible patients

A total of 6037 patients were screened, and 2467 patients reported having visited a clinician at the hospital in the past 2 months. Of these, 923 were current smokers. Finally, 656 currently smoking patients completed the questionnaire and served as our study participants. The screening and enrollment procedures are shown in Figure 1.

### Components of the e-information model

The e-information model was based on a decision support tool embedded in the HIS. This model was developed in reference to the 5 As^5^ and AAR^9^ models in December 2008. After a 3-month pilot testing in the respiratory clinic, the model was officially put into application in all clinics of the study hospital in May 2009. In May 2015, the model was optimized according to feedback from the clinicians. The module operations were simplified, and the contents of the auto-printed information sheet were updated.

**Figure 2:**
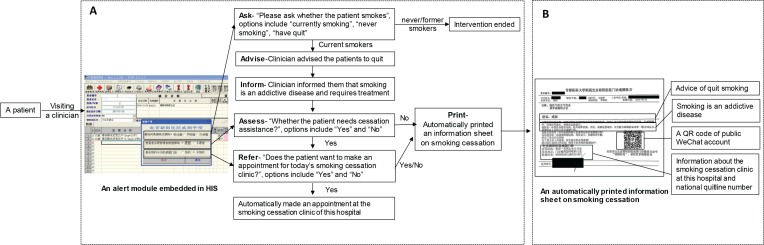
The optimized e-information model

The optimized e-information model had six components (Figure 2A). At the end of the visit, an alert module automatically appeared in the HIS. The clinician was reminded to ask about the patients’ smoking status (Ask) by the first question (‘Please ask whether the patient smokes?’). There were three options: ‘currently smoking’, ‘never-smoking’ or ‘have quit’. If the patient’s smoking status was ‘neversmoking’ or ‘have quit’, the intervention ended. If the patient was identified as a current smoker, the clinician advised the patient to quit (Advise) in a clear, strong, and personalized manner according to the smoking cessation guidelines^5^, and informed them that smoking is an addictive disease and requires treatment (Inform). In China, the proportion of smokers receiving smoking cessation medications is very low (<3%)4, which might be partially explained by a lack of understanding of the nature of smoking addiction by most people in China1. Thus, we designed a novel intervention requiring the clinician to inform currently smoking patients that smoking is an addictive disease (Inform). The transmission of this key information plays an important role in referring smokers to the smoking cessation clinic for treatment. If the response to the first question was ‘currently smoking’, the second question (‘Whether the patient needs cessation assistance?’) automatically appeared, and was used to remind the clinician to assess the patients’ willingness to quit and whether they required cessation assistance (Assess). If the patient was not willing to quit or did not require assistance, the system automatically printed an information sheet on smoking cessation, and the intervention ended. If the patient wanted to quit and required assistance, the third question appeared (‘Does the patient want to make an appointment for today’s smoking cessation clinic?’). If the patient responded no, the system automatically printed an information sheet on smoking cessation, and the intervention ended. If the patient responded yes, an appointment at the smoking cessation clinic of the hospital was automatically made for the patient (Refer). When the operations in this alert module were completed, an information sheet on smoking cessation (Figure 2B) was automatically printed for each identified currently smoking patient (Print). The content of this information sheet was based on the smoking cessation guidelines^5^, including smoking cessation advice, the fact that smoking is an addictive disease, the introduction of effective smoking cessation therapy, information about the smoking cessation clinic of the hospital (including the clinic’s location, experts’ schedules, and telephone number), the national quitline number, and a Quick Response (QR) code for a public WeChat account. By using WeChat app to scan the QR code on the information sheet, patients can obtain more cessation information. Similar to Facebook, Twitter and WhatsApp, WeChat, a free application, has become the most widely and frequently used social networking platform in mainland China. It provides many services^13^ for daily living, including message, free phone calls, interest or private groups, browsing and posting for information sharing, mobile payments etc.

Before the e-information model was applied in 2009 and after it was optimized in 2015, a training session was conducted among clinicians at the hospital, including the introduction of the intervention model and brief smoking cessation intervention skills.

### Measures

A paper questionnaire with 12 closed questions was used to collect the information about participants’ demographics, smoking behavior, self-reported receipt of the components of the e-information model in the past 2 months and their plans to quit within 1 month.

Demographic variables included sex, age, education, and comorbidities. Smoking behavior variables included smoking amount per day and attempts to quit in the past. Considering that smoking is one of the most important risk factors of cardio-cerebral vascular diseases (CCVD) and respiratory diseases, we classified the comorbidities into four categories: CCVD (including coronary heart disease, stroke, and transient ischemic attacks), risk factors for CCVD (including hypertension, diabetes, hyperlipidemia), respiratory diseases (including chronic bronchitis, emphysema, chronic obstructive pulmonary disease [COPD], and asthma), and others (including digestive diseases, cancers, etc.). Participants were asked six questions about their receipt of the components of the e-information model in the past 2 months, including whether the clinicians had asked about their smoking status (Ask), advised them to quit (Advise), informed them that smoking was an addictive disease (Inform), assessed their willingness to quit (Assess), referred them to the smoking cessation clinic of the hospital (Refer), and printed an information sheet on smoking cessation (Print). The participants’ plans to quit within 1 month was assessed by one question: ‘Do you have a plan to quit smoking within 1 month?’.

### Statistical analysis

Participants’ characteristics were described as mean ± standard deviation for continuous variables and as n (%) and 95% confidence interval (95% CI) for categorical variables. The association between the odds ratios (ORs) and 95% CIs of participants planning to quit within 1 month and participants’ receipt of components of the e-information model in the past 2 months was estimated using multivariate logistic regression analysis. The trend test used linear regression to examine differences in participants planning to quit within 1 month by the number of components of the e-information model received. The reference group included participants who received no components of the e-information model. The regression analysis was adjusted by age, education, sex, risk factors for CCVD, and CCVD. All statistical analyses were conducted using SPSS software, version 19.0 (IBM Corp., Armonk, NY, USA). A two-sided p-value <0.05 was considered statistically significant.

## RESULTS

### Participants’ characteristics

Table 1 shows the participants’ characteristics (n=656). Most participants were men (97.4%), were middle-aged and older (mean age: 45.7 ± 14.4 years) and had a high education level (93.0 % had completed senior high school or higher). More than half of the participants (55.0 %) had comorbidities.

**Table 1:** Participants’ characteristics (n=656 )

*Characteristics*	*n*	*%*	*95% CI*
**Sex**			
Male	639	97.4	95.8–98.4
Female	15	2.3	1.3–3.9
**Age** (years)			
<45	322	49.1	45.2–53.0
≥45	305	46.5	42.6–50.4
**Education**			
Less than senior high school	46	7.0	5.2–9.3
Senior high school or higher	610	93.0	90.7–94.8
**Comorbidities**			
Total	361	55.0	51.1–58.4
Hypertension	153	23.3	20.2–26.8
Diabetes	72	11.0	8.8–13.7
Hyperlipemia	92	14.0	11.5–17.0
Coronary Heart Disease	57	8.7	6.7–11.2
Stroke	9	1.4	0.7–2.7
Chronic Bronchitis	25	3.8	2.5–5.6
Emphysema	4	0.6	0.2–1.7
Chronic Obstructive Pulmonary Disease	16	2.4	1.4–4.0
Asthma	32	4.9	3.4–7.0
Digestive tract disease	77	11.7	9.4–14.5
Hepatic-nephrotic disease	9	1.4	0.7–2.7
Cancer	5	0.8	0.3–1.9
Psychological disease	7	1.1	0.5–2.3
Others	37	5.6	4.0–7.7

This study was conducted in the outpatient department of a large urban general hospital in Beijing, China, June–July 2017. We selected participants who were older than 18 years of age, had visited any of the three clinics in the past 2 months, had smoked 100 or more cigarettes in their lives, and currently smoked every day. Patients who reported non-daily smoking or who were unwilling to provide individual demographic information were excluded.

### Self-reported receipt of the components of the e-information model

A high proportion of participants reported having received the Ask (73.2 %) and Advise (65.4 %) components, while a lower proportion reported receiving the Inform (49.8 %) component, and an even lower percentage reported receiving the Refer (16.0 %) and Print (10.4 %) components.

### Association between receipt of the e-information model and participants’ plans to quit

Among the 656 participants, 57.6% reported having a plan to quit within one month. Compared with participants who reported receiving no components of the e-information model, the likelihood of planning to quit among the participants who received the Advise or Inform component increased by 47% (OR=1.47, 95% CI: 1.04–2.08) and 52% (OR=1.52, 95% CI: 1.10–2.11), respectively. After adjustment for sex, age, education, and comorbidities, a dose-response relationship was present between the number of the components of the e-information model received in the past 2 months and the proportion of participants planning to quit within 1 month (p-trend=0.006) (Table 2). The likelihood of planning to quit among participants who had received all five components of the e-information model was highest (OR=2.79, 95% CI: 1.31–5.94). After excluding participants receiving the Refer component, we also found that the likelihood of planning to quit among participants who had received a combination of Ask + Advise, Advise + Inform, Advise + Print, Advise + Inform + Print components increased by 48% (OR=1.48, 95% CI: 1.02–2.15), 64% (OR=1.64, 95% CI: 1.13–2.38), 108% (OR=2.08, 95% CI: 1.14–3.80), and 122% (OR=2.22, 95% CI: 1.19–4.13), respectively (Table 2).

**Table 2:** Association between receipt of the e-information model and participants’ plans to quit^a^

		*n*	*%(95% CI)*		*AOR ( 95% CI)^b^*	*P*
**Components**						
Ask^c^		480	73.2 (69.6–76.5)		1.42 (0.98–2.05)	0.062
Advise^d^		429	65.4 (61.6–69.0)		1.47 (1.04–2.08)	0.028
Inform^e^		327	49.8 (45.9–53.7)		1.52 (1.10–2.11)	0.01
Refer^f^		105	16.0 (13.3–19.1)		1.45 (0.92–2.29)	0.113
Print^g^		68	10.4 (8.2–13.1)		1.70 (0.97–2.98)	0.063
**Number of components**						
None	176			26.8 (23.5–30.4)	Ref.	
Received any 1	38			5.8 (4.2–8.0)	0.88 (0.41–1.85)	0.726
Received any 2	117			17.8 (15.0–21.0)	1.24 (0.76–2.03)	0.384
Received any 3	209			31.9 (28.4–35.6)	1.50 (0.98–2.30)	0.064
Received any 4	70			10.7 (8.5–13.4)	1.30 (0.72–2.33)	0.382
Received all 5	46			7.0 (5.2–9.3)	2.79 (1.31–5.94)	0.008
						p-trend=0.006
**Different combinations of two or three components**						
**Ask + Advise**						
None	176			26.8 (23.5–30.4)	Ref.	
Only Ask	51			7.8 (5.9–10.2)	1.02 (0.53–1.97)	0.946
Ask + Advise	429			65.4 (61.6–69.0)	1.48 (1.02–2.15)	0.040
					p-trend=0.026	
**Advise + Inform**						
None	176			26.8 (23.5–30.4)	Ref.	
Only Advise	115			17.5 (14.7–21.7)	1.22 (0.75–1.97)	0.417
Only Inform	13			2.0 (1.1–3.5)	1.59 (0.50–5.10)	0.436
Advise + Inform	314			47.9 (44.0–51.8)	1.64 (1.13–2.38)	0.009
						p-trend=0.008
**Advise + Print**						
None	176			26.8 (23.5–30.4)	Ref.	
Only Advise	361			55.0 (51.1–58.8)	1.38 (0.97–1.97)	0.073
Advise + Print	68			10.4 (8.2–13.1)	2.08 (1.14–3.80)	0.017
					p-trend=0.010	
**Advise + Inform + Print**						
None	176			26.8 (23.5–30.4)	Ref.	
Only Advise	104			15.9 (13.2–19.0)	1.21 (0.74–1.96)	0.453
Advise + Inform	251			38.3 (34.6–42.2)	1.47 (1.01–2.16)	0.047
Advise + Print	5			0.8 (0.3–1.9)	0.89 (0.12–6.48)	0.905
Advise + Inform + Print	63			9.6 (7.5–12.2)	2.22 (1.19–4.13)	0.012
						p-trend=0.006

a Participants reported they planned to quit within 1 month;

b AOR: adjusted odds ratio by sex, age, education, and comorbidities;

c Ask: ask about the patient’s smoking status;

d Advise: advise the smoking patient to quit;

e Inform: inform the patient that smoking is an addictive disease;

f Refer: refer the smoking patient who needs more cessation assistances to the smoking cessation clinic of the hospital;

g Print: auto-print an information sheet on smoking cessation for each identified smoking patient.

Furthermore, receipt of the Advise component was more likely to be reported by participants aged >45 years (OR=2.0, 95% CI: 1.4–2.8), those with the risk factors for CCVD (OR=2.3, 95% CI: 1.6–3.3), those with CCVD (OR=3.9, 95% CI: 1.8–8.4), and those with respiratory diseases (OR=2.6, 95% CI: 1.4–4.9) (Table 3).

**Table 3:** Receipt of smoking cessation advice among

	*n*	*% ( 95% CI)*	*OR ( 95% CI)*
**Sex**			
Men (n=639)	220	65.6 (61.8–69.3)	Ref.
Women (n=15)	9	60.0 (32.9–82.5)	0.8 (0.3–2.2)
**Age** (years)			
<45 (n=322)	187	58.1 (52.5–63.5)	Ref.
≥45 (n=305)	224	73.4 (68.0–78.2)	2.0 (1.4–2.8)
**Education**			
≥college (n=343)	207	60.3 (54.9–65.5)	Ref.
< college (n=311)	222	71.4 (66.0–76.3)	1.6 (1.2–2.3)
**Comorbidities**			
None (n=295)	150	50.8 (45.0–56.6)	Ref.
Risk factors of CCVD^a^ (n=203)	157	77.3 (70.8–82.7)	2.3 (1.6–3.3)
CCVDs^b^ (n=62)	54	87.1 (75.6–93.9)	3.9 (1.8–8.4)
Respiratory diseases^c^ (n=66)	54	81.8 (70.0–89.8)	2.6 (1.4–4.9)

a Risk factors for CCVD, including diabetes, hyperlipemia, and hypertension;

b CCVD, cardiovascular and cerebrovascular disease, including coronary heart disease, stroke, and transient ischemic attacks;

c Respiratory diseases, including chronic bronchitis, emphysema, chronic obstructive pulmonary disease, and asthma.

## DISCUSSION

The current study investigated the receipt of the e-information model among currently smoking patients in the past 2 months and its effect on patients’ plans to quit smoking within 1 month at a large urban general hospital in Beijing, China. A relatively high proportion of participants reported having received the Ask and Advise components, while a lower percentage reported receiving the Inform component, and a considerably lower percentage received the Refer and Print components. The current findings also indicated that receiving the components of the e-information model may be associated with a higher proportion of participants reporting a plan to quit. To the best of our knowledge, this is the first report on the application and effectiveness of a brief smoking cessation intervention based on HIS in a developing country.

A high proportion of patients reported having received the Ask component, suggesting that the e-information model might prompt clinicians to conveniently identify smoking patients. Identifying and documenting patients’ smoking status are the first two steps to provide smoking cessation interventions and they could support HPs to deliver more cessation assistance^5^. Identifying and recording patients’ smoking status using an electronic approach is time-saving and highly efficient. Consistent with our findings, many previous studies have shown an increase in the recording of the patients’ smoking status (from 37.0–71.6% to 54.0–81.0%) after implementing a brief smoking cessation intervention model based on HIS changes^14-17^. A Cochrane review also demonstrated a similar effect^11^.

We also found that a higher proportion of smoking patients received the Advise (65.4 %) intervention in the current study compared with the China Adult Tobacco Survey (58.2%)^1^. This finding indicates that the e-information model might encourage clinicians to provide smoking cessation advice, which could be related to the reminding function of the alert module embedded in HIS. The findings of several other previous studies are in accord with our results^16,18^. In addition, we found that receipt of the Advise intervention was more frequently reported in patients aged >45 years, those with risk factors for CCVD, those with CCVD, and those with respiratory diseases. These findings are in accord with previous reports that patients with chronic diseases are more likely to have their smoking status recorded and to receive cessation counseling^19^. It is recommended that patients with chronic diseases require more follow-up visits to monitor and manage disease. Thus, such patients more frequently receive cessation interventions from clinicians. Moreover, clinical guidelines on chronic diseases such as COPD^20^, coronary heart disease^21^, and hypertension^22^ recommend that HPs should proactively deliver cessation interventions to smoking patients. Therefore, HPs generally pay more attention to the smoking patients with chronic diseases and are more proactive in providing smoking cessation interventions to them. Consequently, these patients are more likely to recall having received cessation interventions from clinicians.

In contrast to the high proportion of patients receiving the Ask and Advise components, the proportions of patients receiving the other components of the e-information model were relatively low. A previous study reported that only advising smokers to quit did not significantly increase the quit rate^23^. However, higher quit rates could be achieved when quitting advice was combined with other evidence-based cessation assistance, including counseling, prescribing medications and referring patients to cessation services^5^. To support clinicians to provide more cessation assistance in addition to the Ask and Advise components, the e-information model optimized the existing smoking cessation intervention model based on HIS changes^14-17^. In addition to electronic referral to a smoking cessation clinic (Refer), we designed an innovative intervention (Print) to provide cessation assistance to smoking patients. Once a patient was identified as a current smoker, an information sheet on smoking cessation was automatically printed. By using WeChat to scan the QR code on the information sheet, patients were able to obtain more cessation information. This is likely to be more acceptable than traditional paper-based self-help materials because people in China increasingly obtain health-related information through WeChat^13^. In addition, information regarding the smoking cessation clinic and the national quitline number on the information sheet could help clinicians refer smoking patients who need more cessation assistance to professional smoking cessation services. Although the e-information model can be used as a convenient way to help clinicians provide cessation assistance, our results demonstrate that the proportions of patients who actually received the components of the e-information model related to cessation assistance (Refer and Print) were relatively low. This finding is consistent with previous reports that the proportion of referral and medication prescriptions was still low (referral to cessation service: 2.1–15.0%; prescription of cessation medication: 2.5–14.4%) after implementing a smoking cessation intervention model based on HIS changes^14-17,24-28^. Many measures have been proven to effectively motivate HPs to provide cessation interventions, such as payment for performance^29^, regularly delivering feedback on performance^18^, and strengthening training^30^. Based on these measures, the concept ‘system change interventions for smoking cessation’ was proposed, including systematically identifying and documenting patients’ smoking status, strengthening cessation intervention training, providing intervention performance feedback, dedicating special HPs to provide smoking cessation treatment, paying for intervention performance, and providing policy support^31^. These measures could help to improve documentation of patients’ smoking status, provision of cessation counseling, and referral to smoking cessation services^32^.

In addition, we found that the more components of the e-information model the participants received in the past 2 months, the higher was the proportion of participants planning to quit smoking within 1 month. In addition, several previous studies reported that planning to quit is a key predictor of successful quitting^33,34^. Thus, whether the e-information model can increase the quit rate of smoking patients should be verified by large randomized controlled clinical trials in the future.

The EHR system has been used in most hospitals in China^35^, and the effectiveness of the EHR system in China and the United States was found not to be significantly different^36^. Moreover, many other developing countries have already used the EHR system^37^. Therefore, the e-information model including the Ask, Advise, Assess, Inform and Print components, can be directly embedded into the EHR system of a hospital. If the hospital has not set up a smoking cessation hotline or a smoking cessation clinic, smoking patients could be provided with the information via a free hotline, such as the China smoking cessation hotline^12^ or China public service hotline^38^ on the information sheet, or to be referred to other hospitals with a smoking cessation clinic. In addition, we also analyzed the effect of a simplified e-information model composed of two or three components, including Ask + Advise, Advise + Inform, Advise + Print, or Advise + Inform + Print, which also significantly encouraged smoking patients to plan to quit within 1 month. These results demonstrated that the e-information model for a brief smoking cessation intervention based on the EHR system could be also applied in other hospitals in China and other developing countries with or without a simplified model. Given the huge disparities in medical resources between urban and rural in China^39^, the simplified model may be more appropriate in rural clinical settings in China.

### Limitations

The current study involved some limitations. First, our findings were based on patients’ self-reported data, which could lead to recall bias. It is difficult to characterize and quantify potential recall bias, representing a significant methodological limitation. Although it would be better to objectively record the intervention provided by clinicians using the e-information model and related information could be obtained from the EHR system, this possibility may be limited by the functions of the EHR system in the hospital. Moreover, with regard to the high mobility of outpatients and those with chronic diseases re-visiting the clinics once a month based on China’s Medical Insurance System and the needs of chronic disease management, we chose to conduct the current investigation by intercepting patients in the clinic, and focused on patients who had visited any of the three clinics (respiratory, cardiology and neurology), instead of assessing them immediately following their appointment. Second, the current study lacked a control group, and we could not collect the data before and after implementation of the e-information model, which would have provided more convincing evidence. Third, because our sample did not include patients who quit after receiving the e-information model, the effect of the model on the patients’ plans to quit within 1 month may have been underestimated. Fourth, because smoking patients who were not willing to quit did not receive the Refer component, the effect of receiving all components of the e-information model in the past 2 months on the proportion of the participants reporting a plan to quit within 1 month may have been overestimated.

## CONCLUSIONS

The e-information model was applied effectively in a large urban hospital in China, and the results suggest that the model may have increased patients’ plans to quit smoking. These findings indicate that the current approach could be generalized to other hospitals in China. However, many components of this model were less utilized, and comprehensive measures will be required to improve its application in the future.
