# Supplementary material for: Patients’ self-reported receipt of brief smoking cessation interventions based on a decision support tool embedded in the healthcare information system of a large general hospital in China
Source: Tob Induc Dis. 2019 Oct 18;17:73. doi: 10.18332/tid/112567 (PMC6830352; doi:10.18332/tid/112567)
Supplement: Supplementary file 1 [file TID-17-73-s1.pdf]

Description of sample size calculation

The primary outcome of this study was the association of the patients' plan to quit within one month and the components of receiving the e-information model based on multivariate logistic regression models. We assumed there would be about ten independent variables such as age, sex ,education, in the final multivariate logistic regression model and one covariate variables corresponded to 10 positive outcomes(dependent variable, having the plan to quit with one month). Referring to the proportion of smokers having a plan to quit within one year (17.6%) reported in 2015 Global Adult Tobacco Survey, we estimated the proportion of patients having a plan to quit within one month will rise to 35% after receiving the e-information model intervention. Thus, there should be at least (10×10/0.35)=286 patients who would have a plan to quit within one month. To analyze the effect of the e-information model on the patients' plan to quit, we estimated the proportion of smoking patients receiving quit advice from the clinicians after using the e-information model will rise to 65% referring to the data from 2015 Global Adult Tobacco Survey (58.2%)[1]. Therefore, there should be at least (286/0.65)=440 patients who receive the quit advice from the clinicians. A total of 656 subjects were included in this study and were enough to conducted the main analysis.

Reference

1. Chinese Center for Disease Control and Prevention. Global Adult Tobacco Survey (GATS): China 2015 Country Report. Beijing, China: Chinese Center for Disease Control and Prevention; 2015.

© 2019 Chu S.
